# Biogenesis, Features, Functions, and Disease Relationships of a Specific Circular RNA: CDR1as

**DOI:** 10.14336/AD.2019.0920

**Published:** 2020-07-23

**Authors:** Ziyuan Guo, Qidong Cao, Zhuo Zhao, Chunli Song

**Affiliations:** Department of Cardiovascular Internal Medicine, the Second Hospital of Jilin University, Changchun, China

**Keywords:** CDR1as, sponge, miR-7, biogenesis, functions

## Abstract

In 2011, Hansen discovered the natural antisense transcript (NAT) of the cerebellar degeneration-related protein 1 gene (CDR1), and further described CDR1 NAT as a circular RNA (CircRNA). CDR1 antisense RNA (CDR1as), which is the official name of CDR1 NAT, is conserved and extensively expressed in most eutherian mammal brains and other specialized tissues. Further studies have elucidated its biogenesis, features, functions, and relationships with diseases. CDR1as is involved in many disease processes as a microRNA (miR) sponge. Therefore, it seems that further research on CDR1as could facilitate the diagnosis and treatment of some diseases, such as cancer and diabetes. However, a detailed analysis of the results of studies on CDR1as revealed that they are inconsistent and make unclear conclusions. In this review, we gathered and analyzed the recent studies about CDR1as in detail and aimed to elucidate accurate conclusions from them.

As early as 1976, Sanger discovered the existence of circular RNA in plant viroids [1]. However, they were disregarded as rare, wrong, useless splicing products [2,3]. In recent years, the progression of gene sequencing technology and biological computer technology has allowed research on the occurrence, development, and functions of circular RNAs (CircRNAs). Studies of these old, highly stable, highly conserved, non-coding RNAs are revealing interesting details [4,5,6,7]. CircRNAs can act as sponges for microRNAs (miR) and inhibit their functions [5,6,7], miRs are endogenous approximately 23 nt long small RNAs that can base pair to messenger RNA (mRNA) and repress protein production and/or trigger mRNA degradation [8]. Some CircRNAs can be translated into functional proteins [9,10,11,12,13,14], while others can regulate transcription of their host genes [15,16]. However, there is not enough concrete evidence to fully illustrate the powerful functions of CircRNAs [6,7]. CDR1 antisense RNA (CDR1as) is currently among the most extensively studied CircRNAs. In particular, several recent studies have deeply analyzed the functions of CDR1as and its three-dimensional adjustment network with related RNAs, which has allowed more detailed information on CDR1as. In this review, we aim to describe this RNA in detail to allow the reader to have a deeper understanding of CDR1as.

## Biogenesis of CDR1as

In 2011, Hansen first discovered the natural antisense transcript (NAT) of the cerebellar degeneration-related protein 1 gene (CDR1), and further described CDR1 NAT as a CircRNA [17]. When Hansen investigated whether miRs can modulate gene expression by inducing RNAi-dependent transcriptional gene silencing, as done by small interfering RNAs (siRNA), he discovered that miR-671 could direct argonaute (AGO)-2-mediated cleavage of NAT of CDR1 that led to a concomitant decrease in steady-state CDR1 mRNA levels [17]. Next, Hansen went on to characterize CDR1 NAT in more detail, and 3 Rapid amplification of cDNA ends (RACE) analysis failed to reveal any polyadenylation, indicating that the poly(A)-tail is absent in CDR1 NAT [17]. Moreover, he found that CDR1 NAT is resistant to treatment with tobacco acid pyrophosphatase and terminator 5-phosphate-dependent exonuclease, indicating that the 5′-terminal cap structure is also absent in CDR1 NAT [17]. From this, Hansen inferred that CDR1 NAT is a circular exonic RNA produced by non-linear alternative splicing (NAS). Moreover, the strong enrichment of CDR1 NAT in the TRAP fraction (a system that can physically trap circular species upon electrophoresis) relative to the lining RNA layer further confirms its circular nature [17].

**Table 1:** miRs interacting with CDR1as.

Circular RNA	miR	refs
CDR1as	miR-7	8-12, 26, 29, 30, 53-65, 70
	miR-671	8-12
	miR-1299	23
	miR-876-5p	24
	miR-135a	25
	miR-139-3p, miR-471-5p, miR-3065-3p, miR-3132, miR-3134, miR-3145-3p, miR-3201, miR-3617-5p, miR-3637-3p, miR-4254, miR-4291, miR-4306, miR-6807-5p	22

MiRs marked in yellow background may bind to CDR1as, from zhang's prediction by using TargetScan [22], but these have not been proved by experimental studies.

With further research, the role of CDR1as has been gradually explored. Memczak employed sequence analysis across 32 vertebrate species, revealing that human CDR1as harbors 74 miR-7 seed matches and 63 of them are conserved in other species [18]. Hansen also identified 73 conventional seed-targets for miR-7 in the CDR1as sequence, and the binding sites are selectively conserved compared to the adjacent regions in all eutherian mammals, but not in marsupials [19]. Hansen found that CDR1as is globally co-expressed with miR-7 in the brain and acts as a strong sponge for miR-7 [19]. Therefore, Hansen coined CDR1as as ciRS-7 [19]. Is ciRS-7 the proper term for CDR1as? Human CDR1as harbors more than 70 binding sites of miR-7, but none of those sites are complementary with the entire miR-7 sequence [18,19]. The binding sites are complementary only to the 5′ end “seed” region (which is essential for the binding of miR to mRNA) of miR-7 [20]. Instead, CDR1as harbors only one miR-671 binding site, and the site is almost perfectly complementary to the entire mature miR-671 sequence, and miR-671 can directly slice CDR1as [19,21]. A long non-coding RNA (LncRNA) Cyrano harbors a single, nearly perfectly complementary and highly conserved binding site for miR-7, as supported by Cyrano: miR-7 chimeras [20] and Cyrano direct potent, multiple-turnover destruction of miR-7 [21]. In addition, CDR1as also harbors multiple binding sites for other miRNAs [22,23,24,25], as shown in Table 1. Therefore, we do not think that ciRS-7 is a precise term for CDR1as.

**Figure 1.**
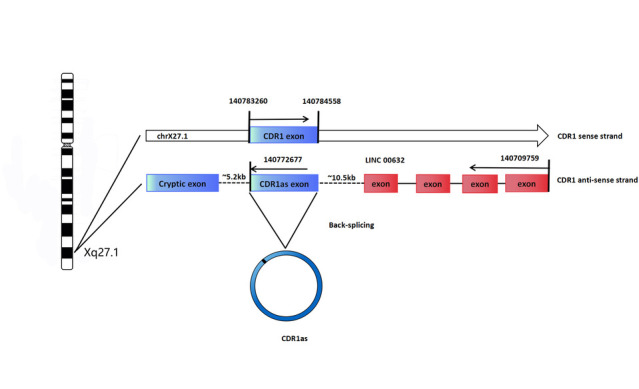
**Model for biogenesis of CDR1as**. In humans, CDR1 gene is located in the sex chromosome site: chrXq27.1 (140783260-140784558), gene ID: 1038 (www.ncbi.nlm.nih.gov/gene/1038), and CDR1 gene is encoded by a single exon without introns [32]. In humans, LINC00632 gene is located in the sex chromosome site: chrXq27.1 (140709759-140772677), gene ID: 286411, and encoded by five exons (www.ncbi.nlm.nih.gov/gene/?term=linc00632). Barrett discovered that the promoters of LINC00632 are responsible for driving CDR1as expression and that the CDR1as sequence is embedded in LINC00632 locus [31]. We speculate the pre-linear RNA of CDR1as is transcribed by the antisense strand of CDR1, which is followed by back-splicing of the 5' and 3' ends to form the circular RNA.

How does CDR1 NAT close into a circle? Generally, CircRNAs are generated when the pre-mRNA back-splice and join a splice donor to an upstream splice acceptor [6,7,26]. Moreover, Alu elements, complementary repeated sequences in the flanking introns, may also be a common driver of back-splicing [6,7,26,27,28,29]. Hansen speculated that CDR1 NAT circularization is mediated by the canonical spliceosomal apparatus that allows 5′-3′ exon-exon linkage [17], which is similar to circular SRY transcription [4] that is also derived from a NAS event [30]. Hansen inserted the CDR1as exon along with the endogenous flanking sequence into pcDNA3 and copied part of the upstream flanking sequence and inserted it downstream in an inverted orientation, but the CDR1as levels remained unaffected [19]. Hansen could not prove the existence of pre-linear RNA of CDR1as, or whether a NAS event occurs in trans, forming antisense concatamers, or whether it is a cis-acting event resulting in a circular NAT [17]. However, Barrett solved this problem when he discovered that the promoters of a LncRNA LINC00632 are responsible for driving CDR1as expression and that the CDR1as sequence is embedded in the LINC00632 locus [31]. In humans, the LINC00632 gene is located in the sex chromosome site: chrXq27.1 (140709759-140772677), gene ID: 286411, and encoded by five exons (www.ncbi.nlm.nih.gov/gene/?term=linc00632). In humans, the CDR1 (also known as CDR34) gene is located in the sex chromosome site: chrXq27.1 (140783260-140784558), gene ID: 1038 (www.ncbi.nlm.nih.gov/gene/1038), and the CDR1 gene is encoded by a single exon without introns [32]. According to Barrett’s research, we can speculate that the pre-linear RNA of CDR1as is transcribed by the antisense strand of CDR1, which is followed by back-splicing of the 5' and 3' ends to form the circular RNA, as shown in Figure 1.

## Features of CDR1as

The length of CDR1as is about 1485 nt in humans and 2927 nt in mice [20]. Although the CDR1as host gene site is located on the X chromosome, qRT-PCR assays of wild-type mice by Piwecka showed similar expression levels of CDR1as in female and male mice, while the CDR1as expression in heterozygous (CDR1as^+/-^) female mice decreased by about half [20]. It is suggested that the host gene of CDR1as on the other X chromosome chain in female mice is inactive, which is in accordance with the theory of X-chromosome inactivation.

Similar to other CircRNAs, CDR1as is also highly conserved and stable, and shows tissue-time specific expression [17, 33, 34]. CDR1as is a single chain circular RNA that lacks a 5' cap structure and 3' poly-A tail structure, and thus cannot be easily digested by the RNA enzyme, RNase II, polynucleotide phosphatase, or oligoribonuclease; therefore, it is more stable than linear mRNAs [17]. Piwecka analyzed CDR1as expression in different tissues of mice, including the cerebellum, olfactory bulb, hippocampus, cerebral cortex, lung tissue, skeletal muscle, spleen, heart, and spinal cord [20]. Moreover, Piwecka found that CDR1as expression is highest in neural tissues, abundant in the spinal cord, low in lung tissue, skeletal muscle, and the heart, and barely detectable in the spleen, indicating a specific tissue distribution pattern of CDR1as expression [20]. The tissue specificity of CDR1as expression was also demonstrated by multiple studies that reported high CDR1as expression in human brain tissue, and no CDR1as expression in unmodified HeLa cells [17, 19, 33]. Barret studied CDR1as expression in HeLa cells, HEK293, and *in vitro* differentiated neuronal cells, and confirmed very low to no CDR1as expression in HeLa cells, moderate expression in HEK293, and very high expression in neurons [31]. At the same time it was found that HEK293 and *in vitro* differentiated neuronal cells are rich in H3K4me3, H3K27ac, and RNA Polymerase II binding sites in the transcriptional start positions of CDR1as [31]. RNA Polymerase II binding, as well as histone modifications H3K4me3 and H3K27ac, are enriched in active promoters, but HeLa cells lack these promoting markers and are rather rich in repressive marker H3K27me3, which play an important role in gene silencing and developmental inhibition regulation [31]. The non-expression of CDR1as in HeLa cells is due to the abundance of H3K27me3, which can induce gene silencing [31]. However, CDR1as expression in HeLa cells significantly increases in the presence of the promoter-activating CRISPRa system and the absence of H3K27me3 inhibition, which indicates an important role of this inhibitor in CDR1as expression [31]. CDR1as expression in mouse cardiomyocytes is not high [20]; however, Geng reported that it significantly increased under acute hypoxia treatment, which suggests that acute hypoxia could regulate CDR1as expression [35]. Liang reported competition between classical splicing and circular RNA formation, and that the lack of core splicing components under pathological conditions may promote circular RNA formation [36]. This is because pre-mRNA preferentially yields circular RNA, rather than canonically spliced linear mRNA, when core spliceosomal components are depleted in cells [36]. However, since CDR1as is a NAT that is encoded by the complementary strand of the CDR1 gene, there is no competition between CDR1as and CDR1 formation, and hence the specific tissue-time distribution pattern of CDR1as expression is not related to this mechanism. Above all, CDR1as expression is not only tissue-time specific but is also regulated by specific stimuli, such as pathological conditions or particular treatments.

## Functions of CDR1as

### Regulation of CDR1 mRNA expression

Hansen employed small internally segmented interfering RNAs (sisiRNA) targeting either the antisense transcript or sense mRNA of CDR1, and found a significant decrease in CDR1 mRNA using either antisense-specific sisiRNA (sisiRNA-AS) or sense-specific sisiRNA (sisiRNA-S) compared with the control [17]. In contrast, only sisiRNA-AS repressed the level of CDR1as, and a significant increase in CDR1as levels was observed after CDR1 mRNA knockdown [17]. Further, Hansen confirmed that CDR1as overexpression promotes CDR1 mRNA expression, but overexpressing the mRNA resulted in decreased CDR1as levels [17]. From this, Hansen proposed a sense-antisense-based feedback mechanism where CDR1as stimulates or stabilizes CDR1 mRNA with a subsequent negative impact on CDR1as levels [17]. Therefore, we can infer that the regulation of CDR1 mRNA expression may be among the ways in which CDR1as exerts its biological role.

### miRNA sponge

At present, many studies have reported that CDR1as performs its biological function mainly by binding and regulating miRs, especially miR-7 [18,19,20,21]. Besides miR-7 and miR-671, CDR1as can also act as a sponge for miR-1299 [23], miR-876-5p [24], miR-135a [25] and play an important regulatory role in tumor development. Zhang [22] also predicted that CDR1as might act as a sponge for about 13 miRNAs, except miR-7 and miR-671, using the TargetScan database (www.targetscan.org/vert_72/). TargetScan predicts biological targets of miRNAs by searching for the presence of conserved 8mer and 7mer sites, which match the seed region of each miR, as shown in Table 1.

Memczak found that the brain tissue of zebrafish embryos is rich in miR-7 but lack the CDR1 gene site and do not express CDR1as [18]. Moreover, the biological phenotype of zebrafish embryos after the injection of plasmid-containing linear version of CDR1as precursor is similar to zebrafish embryos after injection of miR-7 inhibitors, with both having a significantly smaller midbrain [18]. Therefore, CDR1as impairs brain development, similar to miR-7 inhibition, and the injection of the miR-7 precursor can partially improve midbrain volume reduction [18]. This suggests that the biological effect of CDR1as is caused at least in part by the interaction between CDR1as and miR-7 [18]. Moreover, Memczak showed that none of the miR-7 binding sites is complementary beyond position 12 of the miR-7 sequence; the length of the complementary sites is not enough to induce AGO-mediated cleavage of CDR1as. Thus, Memczak concluded that miR-7 can sequester CDR1as but not slice it by AGO-mediated cleavage [18].

Hansen used alkaline phosphatase-coupled probes and found that CDR1as and miR-7 are co-expressed in the brain tissue of mice [19]. The coincidental high expression of CDR1as and miR-7 strongly suggests that miR-7 interacts endogenously with CDR1as [19]. Hansen further assessed the effect of CDR1as expression on miR-7 activity by transfecting miR-7 in a dose-gradient manner, and revealed that established miR-7 targets, such as synuclein alpha, epidermal growth factor receptor (EGFR), and insulin receptor substrate2 (Irs2) [37], responded more efficiently in CDR1as-untransfected control HeLa cells than in CDR1as-expressing HeLa cells [19]. These results suggest that CDR1as inhibits the effect of miR-7 on its target genes [19]. Moreover, the inhibitory effect of CDR1as on miR-7 is significantly weakened after pre-transfection of miR-671 into HeLa cells, indicating that miR-671 indirectly enhances miR-7 activity by inhibiting CDR1as [19]. Hansen proved that miR-671 directs Ago2-slicer-dependent cleavage and removal of CDR1as [17], and that the target sites in CDR1as do not support endo-cleavage by miR-7, but that endogenous CDR1as pull-down by AGO2 is specifically enriched in miR-7-transfected cells compared to controls. Therefore, Hansen speculated that miR-7 can facilitate AGO2 binding with CDR1as and enhance the efficiency of miR-671 to slice CDR1as [19].

Kleaveland reported that CDR1as, Cyrano, miR-7, and miR-671 form a complicated regulatory network in the mammalian brain [21]. First, the expression of miR-7a and miR-7b (in humans, all three miR-7 loci express identical miRs, whereas in mice, the three loci express two variants, miR-7a and miR-7b) in Cyrano^-/-^ mouse cerebellum and hippocampus are significantly increased, and this phenomenon has also been shown in human cells using the CRISPRi system to inhibit the expression of Cyrano. However, primary or precursor miR-7 RNAs and passenger strands are not changed, so Kleaveland concluded that Cyrano promotes degradation of mature miR-7 after it is loaded into AGO. Kleaveland further demonstrated that Cyrano promotes miR-7 degradation by inducing tailing and trimming of miR-7, but tailing appears as a dispensable step of target RNA-directed miRNA degradation [21]. Second, Kleaveland reported that CDR1as expression is significantly decreased in the Cyrano^-/-^ cerebellum, and that this reduction occurred through cytoplasmic destruction rather than reduced production or defective export. Moreover, a similar downregulation of CDR1as expression can be obtained by inducing mutation of the complementary binding site between Cyrano and miR-7. Kleaveland also found that downregulation of CDR1as is not statistically significant after knockout (KO) of the miR-7a and miR-7b gene locus in Cyrano^-/-^ mice. Therefore, Cyrano indirectly promotes the accumulation of CDR1as in brain tissue by promoting the destruction of miR-7 and further inhibiting miR-7-induced CDR1as degradation [21]. Last, Kleaveland generated CDR1as^671-/y^ (miR-671 site within CDR1as is disrupted) mice and found that the CDR1as level is significantly increased in the CDR1as^671-^ mouse brain, proving that miR-671 directs slicing of CDR1as *in vivo*. Kleaveland further generated Cyrano^-/-^; CDR1as^671-/y^ mice, after confirming that miR-7 levels are increased in Cyrano^-/-^; CDR1as^671-/y^ mice compared to CDR1as^671-/y^ mice. Kleaveland assessed CDR1as expression and found that the CDR1as level is decreased in Cyrano^-/-^; CDR1as^671-/y^ mice compared to CDR1as^671-/y^ mice, so Kleaveland speculated that miR-671-directed slicing only partially degraded CDR1as and at least one other unknown miR-7-dependent mechanism acts to degrade CDR1as [21].

**Figure 2.**
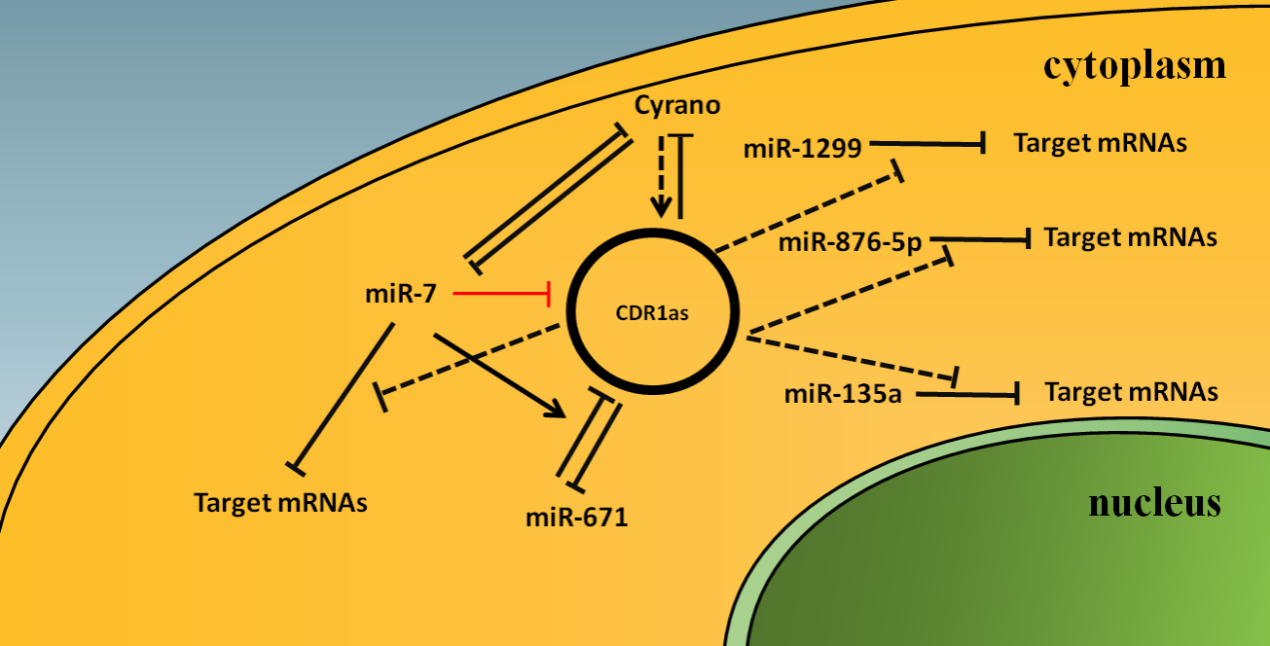
**Model for the network of CDR1as with related RNAs**. CDR1as can act as a sponge for miR-7 and downregulate the inhibitory functions of miR-7 to target mRNAs, but not directly degrade miR-7 [18,19,20], and the same applies to miR-1299 [23], miR-876-5p [24], and miR-135a [25]. When CDR1as is lost, Cyrano is no longer inhibited by CDR1as and upregulated, and miR-7 is no longer sponged by CDR1as and more prone to be degraded by Cyrano [20]. Cyrano indirectly promotes the accumulation of CDR1as in brain tissue by promoting the destruction of miR-7 and further inhibiting miR-7-induced CDR1as degradation [21]. MiR-671 can directly slice CDR1as, while miR-7 can enhance miR-671-directed slicing of CDR1as [19,21] and also act to degrade CDR1as via at least one other unknown miR-7-dependent mechanism [21]. Solid arrow line represents promotion; dotted arrow line represents indirectly promotion; dotted T bars represent inhibition, but not slicing; solid T bars represent degradation; red solid T bar represents degradation, but the mechanism in unknown.

Piwecka obtained hemizygous (CDR1as^-/y^) male mice by KO of the CDR1as gene site using the CRISPR-Cas9 system and found significant downregulation of miR-7 expression and upregulation of miR-671 and Cyrano in the cerebellum, cortex, olfactory bulb, and hippocampus [20]. Further, Piwecka found no significant changes in passenger strand expression of miR-7 and miR-671, which proves that miR-7 and miR-671 are post-transcriptionally deregulated in CDR1as KO brains [20]. Since CDR1as can act as a sponge for both miR-7 and miR-671, the question is why there was specific and opposing deregulation of miR-7 and miR-671 following CDR1as loss? Piwecka explained that miR-671 has one perfectly complementary binding site for CDR1as, which leads to CDR1as slicing and may cause tailing and trimming of miR-671; depletion of CDR1as can induce upregulation of miR-671. In contrast, none of the > 70 miR-7 binding sites has extensive complementarity beyond the seed region, indicating that miR-7 can stably bind but not slice CDR1as [20]. Piwecka speculated that miR-7 decay is promoted and regulated by Cyrano, which is upregulated and can promote miR-7 removal by tailing and trimming in CDR1as KO mice [20].

Therefore, CDR1as can act as a sponge for miR-7 and downregulate the inhibitory functions of miR-7 to target mRNAs, but not directly degrade miR-7 [18,19,20], and the same applies to miR-1299 [23], miR-876-5p [24], and miR-135a [25]. When CDR1as is lost, Cyrano is no longer inhibited by CDR1as and upregulated, and miR-7 is no longer sponged by CDR1as and more prone to be degraded by Cyrano [20]. MiR-671 mainly directs Ago2-slicer-dependent cleavage and removal of CDR1as [17], while miR-7 can enhance miR-671-directed slicing of CDR1as [19,21] and also act to degrade CDR1as via at least one other unknown miR-7-dependent mechanism [21]. We summarized the interaction between CDR1as and related RNAs in Figure 2, but there is a need for more research on the complex adjustment mechanism between CDR1as and related RNAs.

## Relationships with diseases

### CDR1as in disorders of the nervous system

CDR1as can inhibit midbrain development in zebrafish embryos by inhibiting miR-7 [18]. CDR1as expression in Alzheimer's disease (AD) brain tissue is significantly downregulated, and the sponge effects on miR-7 are weakened [38]; therefore, the target genes of miR-7, such as the ubiquitin protein ligase A, are significantly decreased, indicating that the downregulation of CDR1as expression may be related to the occurrence of AD [38]. In CDR1as KO mice, miR-7 expression in the cerebellum, cortex, olfactory bulb, and hippocampus is significantly downregulated, but miR-671, several validated miR-7 targets (such as Fos, Nr4a3, Irs2, Kruppel-like factor-4 (Klf-4)), and Cyrano expression are upregulated [20]. Moreover, there is significant upregulation of immediate early genes (IEGs), such as Fos, Arc, Egr1, Egr2, Nr4a3, and others, which are part of the first wave of response to different stimuli and markers of neuronal activity. Further, upregulation of IEGs is linked to reduced prepulse inhibition (PPI); a PPI deficit manifests as the inability to effectively attenuate the intrinsic startle response to redundant stimuli and clinically correlates with symptoms such as thought disorder and distractibility in schizophrenia [20]. Piwecka also performed an electrophysiological check and found that CDR1as deficiency leads to dysfunction of excitatory synaptic transmission [20]. Given that Piwecka could not rule out CRISPR-Cas9 off-target effects or other unspecific consequences for removing the locus encoding CDR1as, there is a question as to whether these have any effect on the molecular and behavioral phenotype. Piwecka explained that miR-7 and miR-671, which specifically interact with CDR1as, are deregulated in KO mice and there is an upregulation of IEGs, which are linked to the observed neuropsychiatric symptoms and impaired PPI, but not in other tissues with low or no CDR1as expression [20]. As mentioned, Hansen demonstrated a significant decrease in CDR1 mRNA expression using either sisiRNA-AS or sisiRNA-S, and Hansen inferred that CDR1 mRNA expression is dependent on CDR1as level [19]. Therefore, we think that CDR1 mRNA expression should also decrease in CDR1as KO mice. The CDR1 gene is extensively expressed in the brain of humans and mice and plays an important regulatory role in the development of paraneoplastic cerebellar degeneration [32]. So, does decreased CDR1 mRNA expression partly affect the molecular and behavioral phenotype in CDR1as KO mice? Piwecka tried to identify the expression of the CDR1 gene before the generation of CDR1as KO mice, but could not detect any evidence for transcription of the strand opposite to CDR1as in four brain regions in mice using different assays; thus, Piwecka thought that removal of the CDR1as locus is unlikely to have consequences beyond removing CDR1as [20]. That is to say, Piwecka denied the existence of CDR1 in mouse brain, which is inconsistent with the results of Chen [32]. Therefore, there is a need to further confirm whether CDR1 exists and plays a role in neuropsychiatric-like alterations in the behavior of CDR1as KO mice.

### CDR1as in acute myocardial infarction

Acute myocardial infarction (AMI) is myocardial injury with necrosis in a clinical setting consistent with myocardial ischemia. Worldwide, ischemic heart disease is one of most common causes of death and its frequency is increasing [39]. CDR1as may have some regulatory effects on ischemic heart disease [40]. Zhang used whole blood samples to measure the circulating levels of 15 individual LncRNAs and CircRNAs that are known to be relevant to cardiovascular disease, and found that only two of them, a LncRNA ZFAS1 and CDR1as, show significant differential expression between patients with AMI and control subjects. However, although he did not delve into the source of CDR1as in the peripheral blood of patients with AMI, he suggested that the RNA released into the blood is related to the apoptosis and necrosis of cells and/or to cell secretions in microparticles, such as exosomes, microvesicles, apoptotic bodies, and apoptotic microparticles [41]. Geng found that CDR1as and miR-7a are both upregulated in AMI mice with increased cardiac infarct size, or in cardiomyocytes under hypoxic treatment, and that the miR-7a-induced decrease of cell apoptosis under hypoxic conditions can be inhibited by overexpression of the miR-7 target gene SP1 (a transcription factor implicated in hypoxic gene transcription) and poly ADP-ribose polymerase (PARP) [35]. Moreover, *in vivo* overexpression of CDR1as increases the cardiac infarct size and upregulates PARP and SP1 expression, while miR-7a overexpression reverses these changes [35]. However, the increase in endogenous miR-7a expression is not induced by CDR1as overexpression, and miR-7a overexpression alone also has little effect on the apoptosis of normal cultured cardiomyocytes [35, 42, 43].

### CDR1as in cancer

Cancer is the second leading cause of death globally and was estimated to account for 9.6 million deaths in 2018 (www.who.int/cancer/en/). It is currently known that CircRNAs play important regulatory roles in the occurrence and development of tumors [44, 45, 46, 47]. They function in four main ways. First, CircRNAs can compete with the precursor mRNA for splicing and affect RNA splicing to small nuclear RNAs [48]. Second, CircRNAs can enhance the function of the target mRNA through sponge miRNAs [47,49,50]. Third, CircRNAs can be translated into proteins that regulate tumor proliferation under certain circumstances. It was previously thought that CircRNAs cannot be translated into proteins; however, Pamudurti recently reported that CircRNAs can be translated into proteins under certain circumstances [11]. Yang found that the CircRNA circFBXW7 can be translated into a 21-kDa protein, which is termed FBXW7-185aa, the levels of this protein are reduced in glioma, and circFBXW7 expression is positively associated with the overall survival of patients with glioblastoma [12]. Begum reported that circSHPRH can be translated into a tumor suppressor protein, which is associated with survival time of patients with glioblastoma [13]. Gu identified that a CircRNA (hsa_circ_02838) is upregulated in human bladder tumors, and found that hsa_circ_02838 has peptide-coding potential and functions through a peptide-dependent manner; the peptide encoded by hsa_circ_02838 can bind to Gprc5a (a surface protein highly expressed in bladder cancer stem cells, driving bladder tumorigenesis and metastasis) and further enhance the function of Gprc5a to promote bladder cancer; thus, Gu termed hsa_circ_02838 as circGprc5a [14]. Last, CircRNAs can bind to some transcription factors and regulate gene transcription and can also play a tumor regulatory role. For example, Chen reported that circAGO2 derived from the AGO2 gene can regulate the function of AGO2-miRNA complexes, and hence affect the tumor process [51]. However, these studies only reported CDR1as as playing a tumor-regulating role as a miRNA sponge. CDR1as may have effects on tumors via other mechanisms that have not yet been discovered.

**Table 2:** CDR1as in cancer.

CDR1as sponge miR	cancer	target gene	the tumor's progression	Ref
miR-7	BC	CCNE1	promoting	53
	OS	EGFR, CCNE1, PI3KCD,RAF1	promoting	54
	LSCC	proliferation indices ki-67, CCNE1, PIK3CD	promoting	55
	NSCLC	proliferation indices Ki-67, EGFR, CCNE1, PIK3CD	promoting	56
	CRC	EGFR, IGF-1R	promoting	58
	PDAC	EGFR, STAT3	promoting	59
	HCC	EGFR	promoting	60
		CCNE1, PIK3CD	promoting	61
		PIK3CD, p70S6K	promoting	62
	ESCC	KLF-4, NF-κB	promoting	63
		HOXB13-mediated NF-κB/p65	promoting	64
miR-876-5p	ESCC	MAGE-A	promoting	24
miR-1299	TNBC	MMPs	promoting	23
miR-135a	bladder cancer	p21	Inhibiting	25

**Abbreviations:** BC, breast cancer; CCNE1, cyclin E1; CRC, colorectal cancer; EGFR, epidermal growth factor receptor; ESCC, esophageal squamous cell carcinoma; HCC, hepatocellular carcinoma; HOXB13, homeobox gene B13; IGF-1R, insulin-like growth factor-1 receptor; LSCC, laryngeal squamous cell carcinoma; MAGE-A, melanoma antigen gene family-A; MMPs, matrix metalloproteinases family members; NF-κB, nuclear factor kappa B; NSCLC, non-small-cell lung cancer; OS, osteosarcoma; PDAC, pancreatic ductal adenocarcinoma; STAT3, activator of transcription 3; TNBC, triple-negative breast cancer.

Hansen performed a detailed review of the effect of miR-7 on tumors and found that most of the evidence showed that miR-7 has an anticancer effect, but some studies have reported opposite results [52]. Therefore, Hansen mentioned there may be a complex regulatory network regulating the relationship between miR-7 and tumors, and CDR1as may be an important regulatory factor [52]. In recent years, there have been numerous published studies on the association between CDR1as and cancer, as shown in Table 2. Yang found increased CDR1as expression and decreased miR-7 expression in 5-FU-resistant breast cancer (BC) cells [53]. Moreover, Yang demonstrated faster tumor growth in the CDR1as + miR-7 mimic group than in the miR-7 mimic group, and that CDR1as may regulate chemosensitivity in 5-FU-resistant BC cells by inhibiting miR-7 to regulate the miR-7 target gene cyclin E1(CCNE1) [53]. Xu found that patients with osteosarcoma (OS) with large tumor sizes, Enneking stage III, and distant metastasis, had high CDR1as levels but low miR-7 levels [54]. Xu also concluded that CDR1as promotes the progression of OS by inhibiting miR-7 function and upregulating the expression of miR-7 target genes, including EGFR, CCNE1, PI3KCD, and RAF1 [54]. Zhang found that patients with laryngeal squamous cell carcinoma (LSCC) with advanced tumor node metastasis (TNM) stages, poorly differentiated tumors, lymph node metastases, and poor prognosis had high CDR1as levels but low miR-7 levels, and CDR1as promoted the tumor progression by inhibiting miR-7 function and upregulating the expression of miR-7 target genes, including the proliferation indices ki-67, CCNE1, and PIK3CD [55]. Zhang reached a similar conclusion using non-small-cell lung cancer (NSCLC) and found that CDR1as functions as an oncogene that inhibits the anti-tumor effects of miR-7 by upregulation of the proliferation indices Ki-67, EGFR, CCNE1, and PIK3CD [56]. Yan studied disease-free survival and overall survival rates in patients with primary NSCLC who received surgical resection and found elevated CDR1as expression in tumor tissue that positively correlated with tumor size, lymph node metastasis, and TNM stages, and suggested CDR1as high expression as an independent factor for predicting unfavorable disease-free survival and overall survival rates [57]. Tang concluded that CDR1as can partially block miR-7 and positively regulate EGFR and insulin-like growth factor-1 receptor (IGF-1R) to promote colorectal cancer (CRC) progression [58]. Liu also found similar changes in CDR1as and miR-7 expression in pancreatic ductal adenocarcinoma (PDAC) cells, with the final target genes of miR-7 affected by CDR1as being EGFR and activator of transcription 3 (STAT3) [59]. Yang employed a quantitative proteomics strategy to globally identify CDR1as-regulated proteins in hepatocellular carcinoma (HCC) cells and identified 330 differentially expressed proteins upon enhanced CDR1as expression in HepG2 cells, specifically, CDR1as may exert its function by regulating EGFR expression by targeting miR-7 in HCC cells [60]. Yu demonstrated upregulated CDR1as expression and downregulated miR-7 expression in HCC tissues compared with the adjacent non-tumor tissues (ANTs), and knockdown of CDR1as and overexpression of miR-7 can both suppress HCC cell proliferation and invasion by suppressing the direct target gene CCNE1 and PIK3CD expression [61]. However, Xu found no significant difference in CDR1as expression levels between the HCC tissues and ANTs [62]. Xu found that CDR1as expression levels were significantly correlated with the following three clinicopathological characteristics of HCC patients: age < 40 years, serum AFP ≥ 400 ng/µl, and hepatic microvascular invasion (MVI), with CDR1as being among the independent factors of hepatic MVI [62]. Furthermore, CDR1as expression in HCC tissues with concurrent MVI is inversely correlated with that of miR-7 and positively related with that of two miR-7-targeted genes, PIK3CD and p70S6K [62]. Whether the expression level of CDR1as is increased in HCC tissues remains to be further demonstrated, but we believe that the expression level of CDR1as is positively correlated with the degree of malignancy of HCC, similar to other kinds of cancer. The migration and invasion of esophageal squamous cell carcinoma (ESCC) is positively correlated with CDR1as, CDR1as acts as an miR-7 sponge to promote tumor progression by increasing the function of the stem cell marker Klf-4 and homeobox gene B13 (HOXB13)-mediated nuclear factor (NF-κB) [63, 64]. In addition to its role as an miR-7 sponge promoting tumors, CDR1as can also play a role in cancers involving other miRNA sponges. Sang found that CDR1as can act as a competing endogenous RNA of miR-1299 to enhance the expression of matrix metalloproteinases family members (MMPs), and thus maintains the high migration and invasion properties of triple-negative breast cancer (TNBC) cells [23]. Moreover, Sang *et al* also found that CDR1as has 19 binding sites for miR-876-5p and that it accelerates ESCC progression by acting as an miR-876-5p sponge to enhance expression of melanoma antigen gene family-A (MAGE-A) [24]. All the above studies suggest that CDR1as can promote tumors. However, a protective effect of CDR1as on cancer was also reported. Li found that Cdr1as is significantly downregulated and miR-135a is upregulated in bladder cancer tissues compared with ANTs, but the expression of miR-7 is not significantly different [25]. Moreover, Li *et al* found that overexpression of Cdr1as can inhibit the proliferation, invasion, and migration of bladder cancer cells *in vitro* and slow down tumor growth in mice [25]. This anti-oncogenic function is partly via p21 upregulation by sponging miR-135a, but not miR-7 [25]. It seems there is no biological function for miR-7 in the bladder cancer cells, and we speculate this may be related to tissue specificity and low expression levels of miR-7 in the bladder.

In summary, CDR1as can promote the proliferation and metastasis of some tumors by binding and inhibiting miR-7, miR-1299, and miR-876-5p, but CDR1as can also inhibit the proliferation and metastasis of bladder cancer by sponging miR-135a. We hypothesized that the relationship between CDR1as and tumors depends on the type and function of miRs CDR1as sponged in specific tumors. As mentioned above, CDR1as can also sponge many miRs, including miR-671, but there have been no studies on the correlation between CDR1as sponging of other miRs and tumors. Moreover, most of these tumor-related studies are limited to the relationship between CDR1as and a single miR, so cannot further show the complex network between CDR1as, multiple miRs, and other unknown regulatory factors. Therefore, the correlation between CDR1as and tumors is very complex and requires further research.

### CDR1as in diabetes

Xu confirmed CDR1as expression in MIN6 cells (pancreatic β-cell line) and mouse islets and found that CDR1as is significantly increased by several insulin secretagogues such as forskolin and PMA via a forskolin-induced cAMP signaling pathway and PKC signaling pathway, respectively. However, high glucose treatment cannot increase CDR1as expression [65]. Then, Xu *et al* separately transfected miR-7 and CDR1as plasmid DNA into MIN6 cells and mouse islet cells and found that overexpression of miR-7 decreases insulin content and secretion by inhibiting expression of its target genes, Myrip and Pax6, which regulate insulin biosynthesis and secretion, and overexpression of CDR1as can significantly increase the expression of Myrip mRNA and Pax6 mRNA [65]. Therefore, Xu concluded that CDR1as could increase insulin content and secretion by acting as a miR-7 sponge [65]. Stoll searched for circular transcripts expressed in human and mouse islet samples and confirmed expression of CDR1as in insulin-secreting cells, and Stoll reported a reduction in CDR1as levels in both diabetic and obese but still normoglycemic mice [66]. Then, Stoll treated dissociated rat islet cells with CDR1as siRNA for 48 h and observed that insulin secretion tends to be reduced (*p* = 0.058) while the change in insulin content is not statistically significant [66]. However, silencing of CDR1as can diminish prolactin-stimulated proliferation of primary rat β-cells without affecting the survival rate, and the result is similar in MIN6B1 cells using a different siRNA [66]. The negative regulatory effect of miR-7 on β-cell function has been reported [67,68], so we agreed that overexpression of CDR1as can increase the secretion and content of insulin by sponging miR-7. As silencing of CDR1as does not directly regulate the expression of miR-7, it may not affect insulin secretion and content. Moreover, Stoll also reported that silencing of CDR1as reduces proliferation of primary rat β-cells [66], indicating that CDR1as may have other mechanisms regulating the functions of β-cells.

### CDR1as in pulmonary fibrosis

Epithelial-mesenchymal transition (EMT), a process whereby fully differentiated epithelial cells undergo transition to a mesenchymal phenotype giving rise to fibroblasts and myofibroblasts, plays an important role in the process of pulmonary fibrosis [69]. Yao *et al* established the mouse model of pulmonary fibrosis after treatment with silica and found that miR-7 is downregulated in the silica-treated lung tissues of mice, but CDR1as and TGFBR2 (an important receptor of transforming growth factor-β1) are upregulated [70]. Yao also found that the translational activity of the TGFBR2 plasmid is significantly suppressed by miR-7 mimics compared to the binding sites with miR-7 mutant TGFBR2 plasmids and concluded that TGFBR2 is downregulated by miR-7 [70]. Yao found that miR-7 can attenuate silica-induced pulmonary fibrosis by inhibiting TGFBR2, which plays an important role in EMT, but CDR1as can reverse miR-7-mediated repression of TGFBR2. Yao concluded that CDR1as serves as an EMT regulator by suppressing miR-7 in silica-induced pulmonary fibrosis [70]. EMT is integral to development, and the processes underlying it are reactivated in wound healing, fibrosis, and cancer progression. EMT is related to the occurrence and development of various diseases [71]. If CDR1as does play an important regulatory role in the process of EMT, there will be a new field of research on CDR1as.

## Conclusion

Although some research about CDR1as may not be very accurate and needs further and repeated authentication, it is clear that CDR1as, Cyrano, and miRs jointly constitute a complex regulatory network and regulate the function of target mRNA at the post-transcriptional level in cytoplasm. The highest CDR1as expression levels are in brain tissue and abundant in the spinal cord under physiological conditions, low in lung tissue, skeletal muscle, and the heart, and almost non-existent in the spleen. However, the CDR1as expression level is altered in some tumors, pulmonary fibrosis, or myocardial infarction, and we speculate that the regulatory effect of CDR1as is extensive but not specific, as a result, the changes in CDR1as expression are not unique to a single disease; however, CDR1as may be valuable for determining the degree of malignancy and prognosis of tumors. CDR1as can also act as a sponge for miRs and block miRs from binding to their target mRNA, downregulating the inhibitory functions of miRs on their target mRNA. Thus, CDR1as plays an important regulatory role in the pathological process of many diseases, which shows its grander prospect in the field of disease genetic target therapy. We expect more extensive research may finally uncover the true nature and clinical value of CDR1as.
